# Simultaneous Occurrence of Hypospadias and Bilateral Cleft Lip and Jaw in a Crossbred Calf: Clinical, Computer Tomographic, and Genomic Characterization

**DOI:** 10.3390/ani13101709

**Published:** 2023-05-22

**Authors:** Simona Marc, Alexandru Eugeniu Mizeranschi, Cristina Paul, Gabriel Otavă, Jelena Savici, Bogdan Sicoe, Iuliu Torda, Ioan Huțu, Călin Mircu, Daniela Elena Ilie, Mihai Carabaș, Oana Maria Boldura

**Affiliations:** 1 Faculty of Veterinary Medicine, University of Life Sciences ‘’King Mihai I’’ from Timisoara, Calea Aradului 119, 300645 Timisoara, Romania; simona.marc@usab-tm.ro (S.M.); gabrielotava@usab-tm.ro (G.O.); jelenasavici@usab-tm.ro (J.S.); bogdan.sicoe@usab-tm.ro (B.S.); iuliu.torda@usab-tm.ro (I.T.); ioanhutu@usab-tm.ro (I.H.); calinmircu@usab-tm.ro (C.M.); oanaboldura@usab-tm.ro (O.M.B.); 2 Research Institute for Biosecurity and Bioengineering, University of Life Sciences ‘’King Mihai I’’ from Timisoara, 300645 Timișoara, Romania; 3 The Molecular Research Department, Research and Development Station for Bovine Arad, Bodrogului Street, 32, 310059 Arad, Romania; alex.mizeranschi@gmail.com (A.E.M.); danailie@animalsci-tm.ro (D.E.I.); 4 Department of Applied Chemistry and Engineering of Organic and Natural Compounds, Faculty of Industrial Chemistry and Environmental Engineering, Politehnica University Timisoara, Carol Telbisz 6, 300001 Timisoara, Romania; 5 Faculty of Automatic Control and Computer Science, Politehnica University of Bucharest, Splaiul Independenţei 313, 060042 Bucharest, Romania; mihai.carabas@cs.pub.ro

**Keywords:** cattle, cleft lip and jaw, cheilognathoschisis, hypospadias, genomic characterization

## Abstract

**Simple Summary:**

Congenital abnormalities in animals are a major concern for breeders due to the increased economic loss they entail. The aim of this article was to describe a congenital bilateral cleft lip and jaw and an abnormal opening of the penile urethra in a crossbred calf. Clinical examination, computed tomography, and whole genome sequencing were performed to describe and identify a possible cause of the abnormalities. The whole genome investigation indicates the involvement of multiple genes in the two birth defects in this case.

**Abstract:**

Congenital abnormalities in animals, including abnormalities of the cleft lip and jaw and hypospadias have been reported in all domesticated species. They are a major concern for breeders due to the increased economic loss they entail. In this article, we described a congenital bilateral cheilognathoschisis (cleft lip and jaw) with campylognathia in association with penile hypospadias and preputial hypoplasia with failure of preputial fusion in a *Bos taurus* crossbred Piedmontese × Wagyu calf. Clinical examination, computed tomography, and whole genome sequencing were performed to describe and identify a possible cause of the abnormalities. Clinical examination revealed a bilateral cheilognathoschisis of approximately 4 cm in length and 3 cm in width in the widest part, with computer tomography analyses confirming the bilateral absence of the processus nasalis of the incisive bone and the lateral deviation of the processus palatinus towards the left side. Genomic data analyses identified 13 mutations with a high impact on the products of the following overlapped genes: *ACVR1*, *ADGRA2*, *BHMT2*, *BMPR1B*, *CCDC8*, *CDH1*, *EGF*, *F13A1*, *GSTP1*, *IRF6*, *MMP14*, *MYBPHL*, and *PHC2* with *ADGRA2*, *EGF*, *F13A1*, *GSTP1*, and *IRF6* having mutations in a homozygous state. The whole genome investigation indicates the involvement of multiple genes in the birth defects observed in this case.

## 1. Introduction

Congenital oral cavity anomalies are disturbances or failures of the complex processes of craniofacial development. The stomodeum, a primitive oral cavity is formed from five processes: frontonasal, medial nasal, lateral nasal, maxillary, and mandibular, derived from the neural crest mesenchyme and ectomesenchyme [1]. The oral cavity is formed by initial fusions between the medial and lateral nasal processes, followed by fusions between the lateral nasal and maxillary processes, and finally, the maxillary processes merge with the mandibular processes [2,3,4,5].

Failures in the fusion of the lateral and medial nasal processes produce cheiloschisis (lip cleft) and a failure of the medial fusion of the palatal shelves determines palatoschisis (cleft palate). Many molecular signals such as *BMP* (bone morphogenetic proteins), *FGF*s (fibroblast growth factors), *SHH* (Sonic Hedgehog), and *WNT* (Wingless-int) genes, are involved in the growth and fusion of facial processes [6], and mutations of a number of genes, e.g., *BMP4*, *BMPR1A*, *SOX11*, *WNT9B* [7], or *FGF8* [8] affect the normal development of facial processes.

Facial clefts, such as congenital lip and jaw (CLJ), can be classified depending on their location into unilateral right or left side, medial or bilateral CLJ [2,9,10], with a variable expression of the phenotype, both in humans [11,12,13,14] and animals [2,10]. An additional criterion is whether they are associated or not with malformations in other organs: syndromic or non-syndromic forms.

Non-syndromic forms of orofacial defects are classified as cheilopalatoschisis (cleft lip and palate/CLP), only cheiloschisis (cleft lip/CL), or only palatoschisis (cleft palate/CP) [5,14]. They have been reported in cattle [9,10,15], dogs, especially brachycephalic breeds [2], and in horses [16]. Syndromic forms can occur in combination with other developmental disorders such as arthrogryposis [17], anophthalmia, omphalocele, anencephaly, syndactyly, anotia, supernumerary vertebrae, and ribs [2], brachygnathia superior, microcephaly, microphtalmia, and blindness [18].

Possible causes for the non-syndromic forms are multifactorial diseases as a result of the interaction between genetic and environmental factors. They do not follow a mono-genic pattern of inheritance, but they reappear in future generations [2]. Syndromic forms, in many cases, are due to aberrations of chromosomes or are associated with monogenic diseases [19].

Modern genomics techniques have made it possible to identify many candidate genes for CLJ abnormalities in both humans and animals, such as *IRF6* (Interferon regulatory factor 6), *FGFR2* (fibroblast growth factor receptor 2), *SUMO1* (a member of the small ubiquitin-like modifier family), *BMP4* (bone morphogenetic protein 4), *FGFR1* (fibroblast growth factor receptor 1), *MSX1* (MSH homeobox 1), *MAFB* (MAF bZIP transcription factor B), *ABCA4*, *VAX1* (ventral anterior homeobox 1), *WNT* signaling, *FOXE1* (forkhead box E1), *ADAMTS20*, *DLX5*, and *DLX6* [15,20,21,22]. *DLX* genes are essential genes for craniofacial, axial, and appendicular skeletal development; inactivation of *DLX5* and *DLX6* in mice results in severe abnormalities in these regions [23].

In cattle, the frequency of congenital defects is estimated to be 0.25% to 3.6% and can be caused by genetic factors, environmental factors, or genetic–environmental interaction during embryonic development [9,24,25].

Numerous environmental factors have been reported to be associated with facial abnormalities during embryonic development, such as chemicals, toxic substances, infections, hormonal factors etc. [2,4,9].

The other congenital abnormality seen in the calf was hypospadias (OMIA 001187-9913), a developmental abnormality in which the male urethra opens on the ventral side of the penis or on the perineum and can be accompanied by a failed development of the median perineal raphe, and of scrotum, penile aplasia or hypoplasia, hypoplasia of the corpus cavernosum, and incomplete formation of the prepuce [26,27,28]. Abnormal development of the penile urethra is due to a malfusion of the urogenital folds in the midline of normal penile development [26,28]. Depending on where the fusion of the urogenital folds failed, hypospadias can be classified as balanitic/glandular—the urethra opens in the region of the glans penis, penile—the urethra opens ventral and caudal to the glans penis and cranial to the scrotum, and could be proximal, distal, or in the mid shaft of the penis, scrotal—the urethra opens between the halves of the non-fused scrotum or at the scrotum, perineal—the urethra opens in the perineum, ventral to the anus, or anal—the urethra opens in the anal region [24,29,30]. In animals, this abnormality occurs relatively rarely in horses [31], sheep, goats, cattle [24,32,33], dogs, and cats [34,35].

Based on the literature data overview of hypospadias in large and small ruminants, it usually appears in single cases alone with urogenital anomalies (penile aplasia or hypoplasia, preputial aplasia or hypoplasia, cryptorchidism) [25,30,36], or with other abnormalities such as atresia ani, atresia recti, omphalophlebities, growth retardation, and ventricular septal defect [30], atresia of the ear and imperforated left ear [37], anorectal abnormalities, cardiovascular abnormalities, cryptorchidism [36], anuria, hermaphrodism, and rudimentary left kidney and left urethral anomaly [29,30].

In the etiology of hypospadias, there are incriminated genetic, endocrinological, environmental factors and even epigenetic alterations of the genetic material [25,28,38]. Data from the literature indicate that between 10–30% of human hypospadias cases can be at-tributed to specific gene mutations (e.g., AR gene mutation, SRD5A type II deficiency), while 70% of cases have unknown etiology, possibly a combination of genes and environmental factors [38,39,40]. Abnormal expression patterns of some transcription factors, with a key role in the spatiotemporal regulation of gene expression, may disrupt the biological processes during embryogenesis [41].

The morphogenesis of the genital tubercle involves different genes such as *BMP2*, *BMP4*, *BMP7*, *FGF*, *FGF10*, *FGF8*, *HOXA13*, *HOXA4*, *HOXB6*, *HOXD13*, *TGF*, and *WNT5A* genes [41,42,43]. Moreover, *ESR1* and *ESR2* genes through estrogens play a role in normal penile and clitoral development, and normal urethral development depends on the balance between androgens and estrogens [44,45,46]. Studies carried out on mice showed that the exposure of pregnant females to external estrogens induced a thin periurethral spongiosa in the offspring [47] and the loss of endogenous estrogen signaling in mice can cause a mild hypospadias in the offspring [44].

Additionally, epigenetic alterations can be involved in hypospadias development such as the methylation of the *AR* gene [48], the *SRD5A2* gene [49], or CpG sites [28], determining the use of DNA methylation patterns to identify and evaluate new candidate genes that may be involved in the etiology of hypospadias [28].

The purpose of this study was to investigate a male Piedmontese × Wagyu calf with congenital bilateral cheilognathoschisis and hypospadias using computer tomography analysis and whole genome sequencing in order to identify the possible cause of those congenital defects. To our knowledge, based on literature data, we present a unique case of congenital defects in a *Bos taurus* hybrid Piedmontese × Wagyu crossbred cattle using different paraclinical examinations.

## 2. Materials and Methods

### 2.1. Statement of Ethics

The case study was performed in compliance with national (Romanian Law 43/2014) and European guidelines (EU Directive 63/2010) for using animals for scientific purposes, and in accordance with the internal SOP (Operational Procedures System) of USAMVBT-PG-001-R021 (Banat’s University of Agricultural Sciences and Veterinary Medicine) and Ethical Statement no. 73/2020.

### 2.2. Animal

The male *Bos taurus* hybrid Piedmontese×Wagyu crossbred calf with a body weight of 32 kg was born alive after a normal pregnancy period of a heifer in September 2020, in the Didactic Farm of the University of Life Sciences ‘’King Mihai I’’ from Timişoara, Romania. Its mother was a primiparous Piedmontese crossbred (a product between a Holstein female and a Piedmontese male) and the father was from the Wagyu breed. It was the first case with such congenital abnormalities in our Research Farm and, to our knowledge, the first such documented case in the world. The calf was euthanized a few days later due to severe neonatal diarrhea and anemia, as clinically it was impossible to feed properly with colostrum and it could not be kept in a quadrupedal position. Euthanasia was performed with Ketamidor 100 mg/mL from Richter Pharma, Austria, and Xylazin Bio 20 mg/mL from Bioveta, Cech Republic and followed by T61 (embrutramide 200 mg/mL, mebezonium iodide 50 mg/mL, tetracaine hidrochloride 5 mg/mL) 1 mL/10 kg, intravenous, from Intervet International BV Wim de Korverstraat, Holland.

### 2.3. Computer Tomography (CT) Analyzes

The acquisition of CT scan images was performed postmortem with the body positioned such that the ventral aspect of the head was in contact with the CT table (ventral recumbency). CT scans were performed with a Siemens Somatom Definition AS 64 scanner, using conventional settings (90 kV, 100 mAs), and a slice thickness of 0.6 mm.

### 2.4. Whole Genome Sequencing

Genomic DNA was isolated from whole blood collected from the affected calf using a DNeasy Blood & Tissue Kit (Qiagen, Hilden, Germany) for DNA extraction. Whole-genome sequencing was performed by Novogene, Cambridge, United Kindom. The sample was 41.13 µL in volume, 10.209 ng/µL concentration with a total amount of DNA of 0.41989 µg. The DNA concentration was measured using a Qubit^®^ DNA Assay Kit on a Qubit^®^ 2.0 Fluorometer (Life Technologies, Carlsbad, CA, USA).

Next, the library preparation was performed. Briefly, the genomic DNA was randomly fragmented into 350 bp fragments, and then DNA fragments were end polished, A-tailed, and ligated with the adapters for Illumina sequencing, and further PCR enriched with P5 and P7 oligo primers. The PCR products as final construct of the libraries were purified (AMPure XP system), followed by a quality control test that included the size distribution using an Agilent 2100 Bioanalyzer (Agilent Technologies, Santa Clara, CA, USA) and the measurement of molarity using real-time PCR.

Raw sequenced reads in FASTQ format were analyzed using the Bcbio-nextgen pipeline v. 1.2.9. The quality control of the raw data was performed with FastQC v. 0.11.p [50]. Read decontamination was performed with kraken2 v. 2.1.2 [51] using the options “--minimum-hit-groups 4 --confidence 0.5” and a newly-created database consisting of all the genomic sequences found in the NCBI RefSeq database [52] as of May 2022 for human, bacteria, archaea, viruses, fungi, and protozoa. The databases consisted of 101,535 sequences with a summed length of 138.7 Gbp and occupied approx. 117 GB of storage space. The decontaminated reads were mapped to the ARS-UCD1.2 reference genome [53] using the BWA software package v. 0.7.17 [54]. The FASTA file with genomic sequences and the corresponding GTF file with genomic locations for the ARS-UCD1.2 reference were acquired from Ensembl, release 105 [55]. The variant calling was performed with GATK HaplotypeCaller v. 4.2.6.1 [56]. Low-complexity regions consisting of microsatellites and simple repeats were downloaded from UCSC for the ARS-UCD1.2 reference and excluded from the variant calling process. Variant effects on gene transcripts were predicted using the Ensembl Variant Effect Predictor (VEP) tool v. 105.0 [57] and the Ensembl GTF file mentioned above.

The variants classified as having a high or moderate impact and the corresponding genes were retained for the downstream analysis, which was performed using various packages for the R programming language v. 4.1.3 [58]. A list of 126 genes implicated in hypospadias was put together following a survey of the relevant literature, as previously described [27,30,40,59]. Similarly, a list of 513 genes known to be involved in the occurrence of cheilognathoschisis (cleft lip and jaw) was obtained from the CleftGeneDB database [60], as of July 2022. After eliminating duplicate genes that were associated with hypospadias and cleft lip and jaw, a set of 559 unique target genes was retained.

Finally, gene annotations were obtained via the AnnotationHub package v. 3.2.2 [61] and multiple over-representation analyses based on GeneOntology (GO) [62], Kyoto Encyclopedia of Genes and Genomes (KEGG) [63], Medical subject headings (MeSH) [64], and Reactome [65] were performed with the R packages clusterProfiler v. 4.2.2 [66], meshes v. 1.20.0 [67], and ReactomePA v. 1.38.0 [68]. In order to perform the previously mentioned over-representation analyses, common gene identifiers reported by Ensembl VEP for high impact and moderate impact variants were searched within the corresponding OrgDb and MeSHDb annotation databases from AnnotationHub for *Homo Sapiens*, using the *select* function from the AnnotationDbi package v. 1.56.2 [69]. Gene annotation was performed on sets of all genes associated with high-impact and/or moderate-impact variants (i.e., not only for the 559 target genes), in order to find significantly associated annotation terms with the specific variants found for the *Bos taurus* individual from this study.

## 3. Results

### 3.1. Clinical Findings

**Figure 1:**
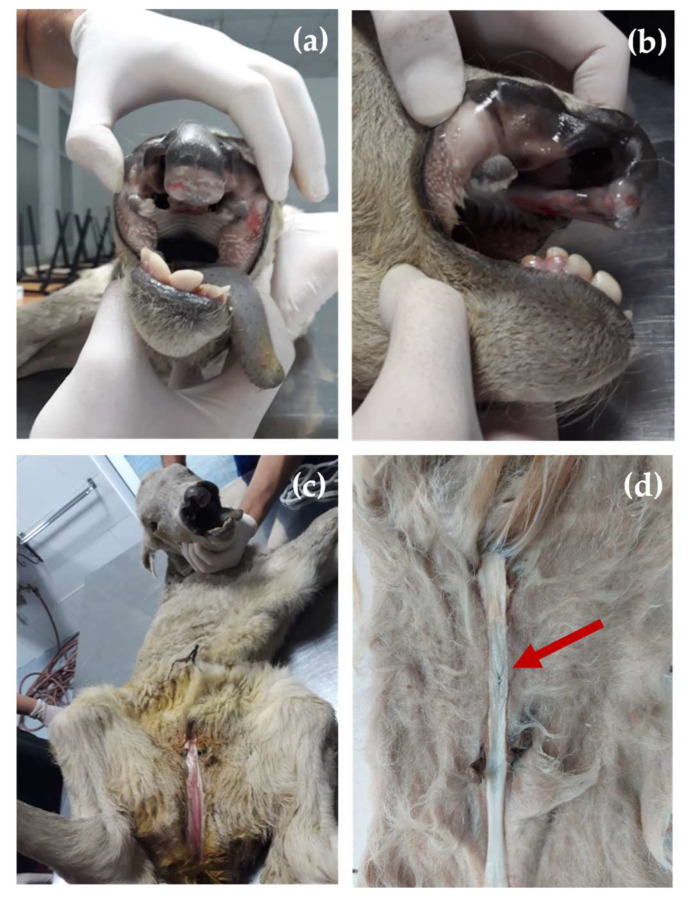
Clinical aspects of the calf: (**a**) frontal view of the cleft lip and jaw; (**b**) lateral view of the cleft lip and jaw; (**c**) large hypospadias of the calf; (**d**) opening of the urethra pars penina (red arrow).

At birth, the affected calf presented a cleft lip and jaw (OMIA 001714-9913) and hypospadias (OMIA 001187-9913). The bilateral cleft was characterized by a length of approximately 4 cm on each side and a width of 3 cm on the widest part. On the andrological examination, the calf showed penile hypospadias, the penile urethra ventrally opened at approximately 4 cm caudal of the normal opening of the urethra and the prepuce was incompletely developed and had failed to fuse normally (Figure 1a–d).

The anus was open physiologically. The examination of the scrotum revealed that the testicles were not fully descended and at the necropsy, the testicles with a length of 2.2 cm were identified in the inguinal canal. The necropsy revealed that there was no other internal congenital abnormality in addition to the previously mentioned external defects.

### 3.2. Computed Tomography Findings

**Figure 2:**
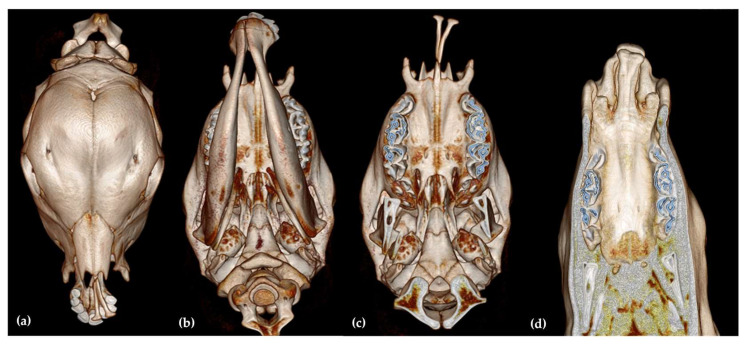
Computed tomography 3D reconstructions of the skull, including the mandibles, revealing a complete bilateral CLJ (**a**) dorsal view, (**b**) ventral view, excluding the mandibles, from a ventral view (**c**), computed tomography 3D reconstruction of the head, excluding the mandibles, from a ventral view (**d**). Note the absence of the lateral aspect of the dental plates bilaterally.

CT of the head (Figure 2) revealed a bilateral cleft lip and jaw, with the bilateral absence of the processus nasalis of the incisive bone (Figure 2a–c). Only the processus palatinus was identified as being completely developed bilaterally, but both were deviated laterally toward the left side. A complete absence of the lateral aspects of the dental plate was observed bilaterally (Figure 2d).

Based on cleft lip-jaw scoring system in cattle existing in literature where different orofacial structures, such as lips, the processus nasalis of the os incisivum, the dental plate with adjacent segments of the hard palate, the facial bones—os incisivum, os maxillare, os nasale, os palatinum, and the mandibles, we scored the cleft lips and accompanying malformations in the face in this case as fallow. The score was 4 (on a scale from 1–4) for the site (bilateral left and right); a score of 5 (on a scale from 0–5) for the depth of the cleft lip (complete cleft of the upper lip independent of whether the maxillary alveolus is affected), a score of 6 (on a scale from 1–7) for changes at the dental plate and hard palate (about 2/3 of the dental plate and adjacent segments of the hard palate were missing), a score of 1 (on a scale from 0–3), for curvature to the right (lateral curvature of the mandibles), and a score of 5 (on a scale from 0–9) for changes in the processus nasalis of the os incisivum (absence of the apical segment of the processus nasalis).

### 3.3. Whole Genome Sequencing Findings

Statistics regarding the proportion of reads mapped to the reference genome were computed. There was a total of 164.9 million mapped reads, a proportion of 98% of the total of reads. The percentage of PCR duplicate reads was 29%. The percentage of 1X coverage was 93.9%, for 5X coverage was 45%, for 10X coverage was 14.1%, with the mean genome coverage of 6.3X, as shown in Supplementary Figure S1.

**Figure 3:**
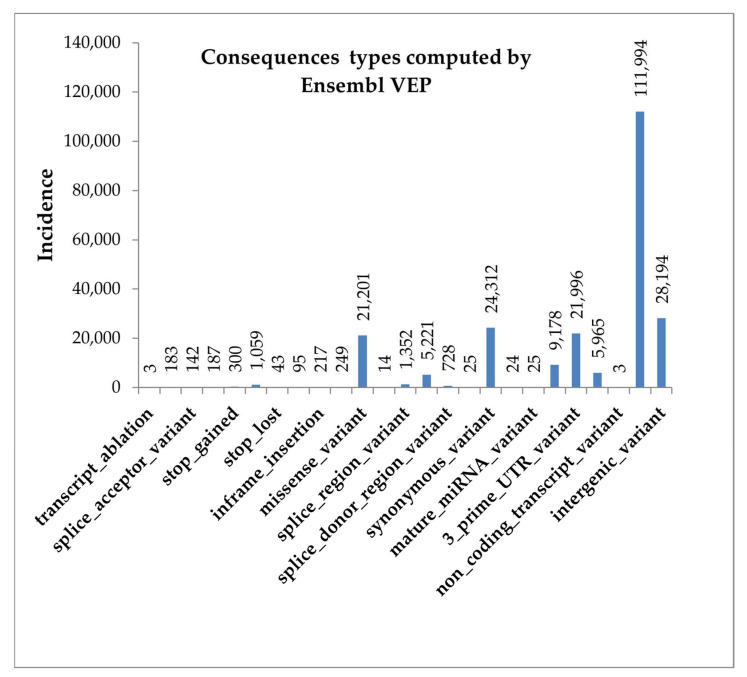
The incidence of most severe consequence types per variant, as computed by Ensembl VEP.

Variant calling identified a total number of 5,598,215 variants, of which 4,951,834 were SNPs and 648,459 were indels. Predicted consequences of all variants (most severe per variant), computed by Ensembl Variant Effect Predictor (VEP), are presented in Figure 3, with the exception for upstream-gene-variants, which were 3,525,429, and intron-variants with 1,840,076 incidence.

Most of the variants, such as non-coding transcript variants, intergenic variants, synonymous variants, are considered as unlikely to change protein behavior or with low or no impact on it. Conversely, as can be seen in Figure 3, Ensembl VEP identified a low number of variants that are assumed to have a high impact in the protein (transcript ablation, splice acceptor variant, stop gained, or stop lost).

An initial quality control checks on raw sequenced reads using FastQC revealed one “failure” result for the “Per sequence GC content” test, indicating a possible contamination with DNA from a different species. Subsequently, this scenario was checked with the Kraken2 tool, which classified 21.97% of the original raw reads as one of the taxons present in the reference database, consisting of all humans, bacterial, archaeal, viral, fungal, and protozoan sequences from the NCBI RefSeq database as of May 2022. Of the remaining, unclassified reads, which amounted to 164.9 million reads, 161.6 million reads (98%) mapped to the bovine reference genome ARS-UCD1.2.

**Figure 4:**
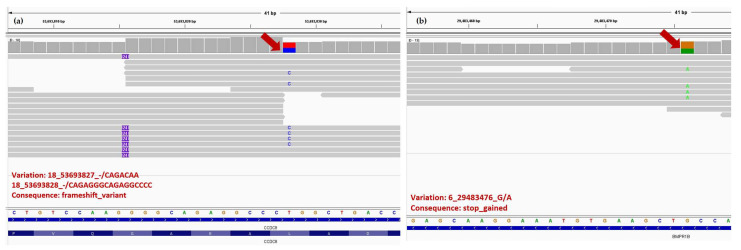
The IGV images of new *CCDC8* (**a**) and *BMPR1B* (**b**) high impact target gene variations and with their gene structure consequence that were not reported in literature with implication in humans or animals on CLJ or hypospadias, except for one study [30]. The positon in reads is indicated with red arrows, which contains the mismatches with the reference. In *CCDC8* gene, the figure depicts an insertion of 24 nucleotides.

**Figure 5:**
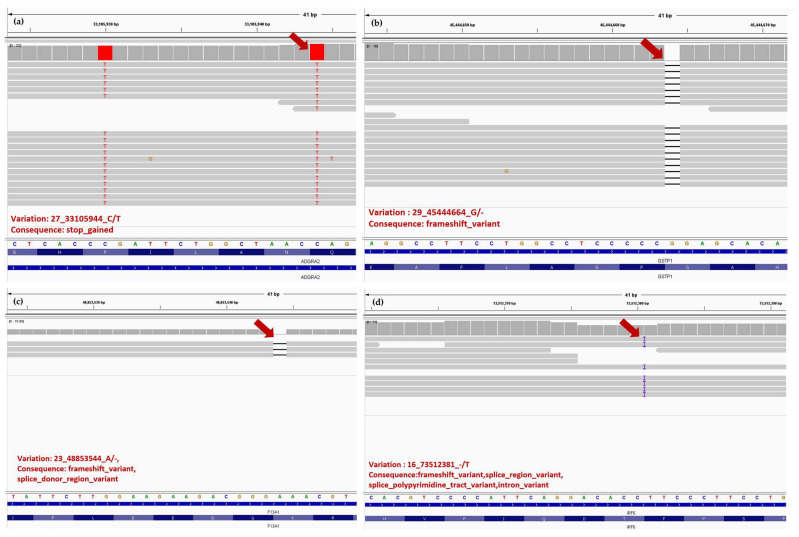
The IGV images of four high impact target gene variations in exome from the sample and with their gene structure consequence for the gene structure, where (**a**) *ADGRA2*, (**b**) *GSTP1*, (**c**) *F13A1*, (**d**) *IRF6*, and the position of variations in reads is indicated with red arrows.

IGV (integrative genome viewer) images for *ADGRA2*, *BMPR1B*, *CCDC8*, *F13A1*, *GSTP1* and *IRF6* genes are presented in Figure 4 and Figure 5.

The raw sequencing data have been uploaded to the NCBI SRA database under the accession number PRJNA896881.

The list of 559 unique bovine genes that were found associated with hypospadias and CLJ in cattle and other mammals, according to literature and the CleftGeneDB database, are presented in an Excel sheet (Supplementary S1 file). The chromosomal positions are those corresponding to the ARS-UCD1.2 *Bos taurus* reference genome. Supplementary S1 file also contains a list of 379 genes associated with high-impact variants and the list of 4049 genes associated with moderate-impact variants.

Table 1 presents the variant annotation genes known to be involved in CLJ and hypospadias in mammals with a high impact on the following overlapped genes: *ACVR1*, *ADGRA2*, *BHMT2*, *BMPR1B*, *CCDC8*, *CDH1*, *EGF*, *F13A1*, *GSTP1*, *IRF6*, *MMP14*, *MYBPHL*, and *PHC2*.

The list of SNPs reported by Ensembl VEP as having a high impact on any of the 559 target genes are presented in an Excel sheet (Supplementary S2 file) and the list with those having a moderate impact on any of the 559 target genes are presented in the Excel sheet named Supplementary S3 file.

Gene annotations based on GO, KEGG, MeSH, and Reactome, which were found significant after over-representation analyses based on genes with moderate and high impact SNPs (379 and 4049 genes, respectively), are included in Supplementary S4 file, with the corresponding sheet names following: *GO_high_moderate* = Significant Gene Ontology terms for genes overlapped by variations with a high or moderate effect; *KEGG_high_moderate* = Significant KEGG terms for genes overlapped by variations with a high or moderate effect; *MeSH_high* = Significant MeSH terms for genes overlapped by variations with a high effect; *MeSH_high_moderate* = Significant MeSH terms for genes overlapped by variations with a high or moderate effect; *Reactome_high_moderate* = Significant Reactome terms for genes overlapped by variations with a high or moderate effect.

The biological terms and pathways in the gene annotation databases consist of 5 GO terms, 2 KEGG pathways, 12 MeSH terms, and 5 Reactome pathways. A total of 490 genes were assigned to the Biological Process (BP) GO term category (3 terms), and 188 genes to the Molecular Function (MF) GO term category (2 terms). The analyses based on GO were highly enriched in genes associated with sensory perception of smell (GO:0050907, GO:0050911, GO:0007608), olfactory receptor activity (GO:0004984), cargo receptor activity (GO:0038024), olfactory transduction, and glycosaminoglycan biosynthesis—chondroitin sulfate/dermatan sulfate (KEGG).

MeSH analyses captured several significant terms such as Gamma Catenin (MeSH:D051185), Glyoxal (MeSH:D006037), Sertoli Cell Tumor (MeSH:D012707), Desmogleins (MeSH:D051182), Factor VIII (MeSH:D005169), Hair Cells and Auditory (MeSH:D018072), Cytokinesis (MeSH:D048749), Glycopeptides (MeSH:D006020); Desmocollins (MeSH:D051187), Lung Diseases (MeSH:D008171). Several of these MeSH terms are associated with *TF* (transferrin), *EGF* (epidermal growth factor), *MMP14* (Matrix metallopeptidase 14), *CDH1* (Cadherin 1), and *F13A1* (Coagulation factor XIII A chain) genes, related to CLJ and hypospadias.

Reactome pathway annotations uncovered the following five significant terms: Olfactory Signaling Pathway (R-HSA-381753); Degradation of the extracellular matrix (R-HSA-1474228); Defective B3GALTL causes Peters-plus syndrome (PpS) (R-HSA-5083635); Diseases of metabolism (R-HSA-5668914); and O-glycosylation of TSR domain-containing proteins (R-HSA-517321). Notably, the latter four terms included numerous genes from the *ADAM* family (Supplementary S4 file).

## 4. Discussion

Cleft lip and jaw and hypospadias are two congenital malformations which appear in populations due to various gene mutations, environmental factors, or both. The molecular mechanisms by which the two malformations appear are very complex and different. Among the genes known to be involved in cleft lip and jaw, we mention *ABCA4*, *ADAMTS20*, *BMP4*, *BRD1*, *CREBBP*, *CSK*, *DLX5*, *DLX6*, *DNM1L*, *FGFR1*, *FGFR2*, *FOXE1*, *IRF6*, *LOR*, *MAFB*, *MSX1*, *PTPN18*, *SND1*, *SUMO1*, *TGS1*, *VAX1*, *VIM,* and *WNT* [23,70]. For hypospadias, also the molecular mechanism is complex, many genes are implicated (e.g., *AR*, *ATF3*, *ATF3*, *BMP4*, *BMP7*, *BNC2*, *CTGF*, *CYP1A1*, *CYR61*, *DGKK*, *EGESR1*, *ESR2*, *FGF8*, *FGFR2*, *GSTM1*, *GSTT1*, *HOXA4*, *HOXB6*, *HSD17B3*, *HSD3B2*, *MAMLD1*, *MID1*, *SF1*, *SRD5A2*, *WT1* [28,38,59,71,72]; *CYP11a1*, *CYP17a1*, *HES3*, *HSD3b1*, *INSL1*, *SF1*, *STAR*, *STAT3* [73], *DGKK*, *STS*, *ZEB1* [27], *EFNB2* [40], or *NR5A1* [74]), genes that physiologically are involved in the development of the male external genitalia in an indifferent stage or in the hormone-dependent stage.

During CT analysis of this case, high scores were observed for orofacial defects according to the cleft lip and jaw scoring system developed by Reinartz et al. [10] in cattle. The right campylognathia seen in this case may be a consequence of the absence of the pressing surface between the mandible and the missing segments of the os incisivum contralateral to the cheilognathoschisis.

Following the analysis of the genomic sequences, the genes with high, high/moderate, and moderate impact on the two congenital diseases were identified, as well as variations of these genes with high or moderate impact. We identified a large number of genes, 379 genes with 1103 genetic variations that can have a major impact on the phenotype. The genes overlapped by variations with high impact or moderate impact on all genes were in the number of 4177. and 221 were with impact on genes of interest, with 16,026 variants with high or moderate impact on all genes, as well as 4049 genes overlapped by variations with moderate impact, having 14,924 variations with moderate impact on the genes on which they were overlapped.

Among them, whole genome sequencing and variant annotation focused on genes known to be involved in CLJ and hypospadias in mammals identified 13 mutations with a high impact on the following overlapped genes: *EGF*, *IRF6*, *BMPR1B*, *MMP14*, *ADGRA2*, *F13A1*, *CCDC8*, *ACVR1*, *BHMT2*, *CDH1*, *GSTP1*, *PHC2*, and *MYBPHL* where the *EGF*, *IRF6*, *F13A1*, *ADGRA2*, and *GSTP1* genes were found with homozygous mutation, and the *CCDC8* and *BMPR1B* genes were not reported in the literature (except [30]) with implication in humans or animals CLJ or hypospadias, nor did the two genes in the Cleft Gene DataBase.

For the EGF gene (ENSBTAG00000048812), the identified mutation located in the position 6:g.15450370G>A (p.Arg105Ter) revealed a homozygous single nucleotide variant, and the consequence was a stop gained (premature stop codon). The protein encoded by EGF gene has 232 amino acids and the affected amino acid is located at position 105. *EGF* (epidermal growth factor), a prototypical ligand, and epidermal growth factor receptor (*EGFR*) are part of the *EGF* family, being important for cell motility, adhesion, proliferation by enhancing cell cycle progression through the G1 phase, differentiation, and survival being ubiquitously expressed in somatic tissues and in the gonads [75]. *EGFR* has an important role in normal craniofacial development, *EGFR*-/- with decreased matrix metalloproteinases (MMPs) in mice embryos induced facial anomalies (hypognathia, lack of eyelids, cleft palate) [76]. *MMPs*, part of a protease family that comprehends adamalysins (*ADAMs*), disintegrin, and metalloproteinase with thrombospondin motifs (*ADAMTSs*), with an important role in extracellular matrix remodeling, morphogenesis, have a possible implication in the etiology of CL/P [77]. A correlation between the low expression level of *EGF*, *EGFR*, and *HOXA13* in the foreskin tissue and hypospadias cases indicates a possible cause of hypospadias [78].

Another gene identified as having a potential high risk was the *BMPR1B* gene (ENSBTAG00000002081) (Supplementary S2 file), a member of BMP cytokins. The heterozygous mutation identified in the BMPR1B gene was 6:g.29483476G>A (pGln10X) and the consequence was the introduction of a premature stop codon. Studies indicate that BMP signaling molecules are involved through active *ACVR1* (activin A receptor type 1) in the palate epithelium and an increase of this signaling pathway causes a submucous palate [79]. BMPs, cytokines that control the function of many types of cells, exert their role through *BMPR1A*, *BMPR1B*, and *BMPR2* class receptors with many other receptors in each class, and loss of function can be associated with muscular-skeletal and, cardiovascular diseases, cancer [80], or CP [81]. BMP and growth factors, including *EGF*, play the role of paracrine mediators for epithelial–mesenchymal interactions during the embryonic development of various organs, including the male reproductive system, *EGF* stimulating androgen binding activity in the male genital tract, very important hormones for the fetal development of the reproductive system during the hormone-dependent period [40,42,82]. Mutation of *BMP4* and *BMP7*—two factors that play a role in normal urethra development, along with *HOXA4* and *HOXB6*, were identified in Chinese patients with hypospadias, but without a clear association [83].

For the *IRF6* gene (ENSBTAG00000002849), the homozygous state mutation identified was 16:g73512380-73512381insT, a frameshift variant consisting of an insertion of a nucleotide at position 162. The *IRF6* gene (Interferon Regulatory Factor 6) plays an important role in normal early development. Its function is active in the cells from which the tissues of the face, head, skin, and genitals are formed. In the normal development of the epidermis, *IRF6* regulates the balance between keratinocyte proliferation and differentiation, as well as in craniofacial morphogenesis, oral epithelium and breast epithelium [84,85].

Different mutations in the *IRF6* locus may increase the risk of syndromic and nonsyndromic forms of orofacial clefts; in humans, for example, mutations in the *IRF6* gene are associated with Van der Wounde Syndrome (VWS) (OMIM # 119300) and Polipteal Pterygium syndrome (OMIM # 119500), rare orofacial cleft syndromes associated with lip pits, and cutaneous and limb defects [86,87]. In approximately 70% of VWS cases, mutations in the *IRF6* gene are responsible for the disease and Zang et al. (2020) identified a frameshift mutation in *IRF6* as a possible cause (NM.006147.3, c.1088-1091delTCTA; p.Ile363ArgfsTer33) [88]. Moreover, the *IRF6* gene variations are believed to affect the function of the IRF6 protein in its role as a transcription factor, which may interfere with the normal development of the face. Keratinocyte deficiency in *IRF6* affects intracellular adhesion and cell colony morphology, an aspect observed clinically postoperatively in patients with cleft-lip/cleft palate by delaying wound healing [85]. An *IRF6* SNP may play a major role in the pathogenesis and risk of developing non-syndromic cleft lip +/− palate in a South Indian population [89]. Loss of *IRF6* in mice leads to skin, craniofacial, and limb defects. A minimum level of *IRF6* in the periderm and basal epithelial cells is necessary for orofacial development and a partial genetic rescue of the *IRF6* knockout embryo with the *KRT14* promoter can be performed [90].

The *ADGRA2* gene (adhesion G protein-coupled receptor A2)(ENSBTAG00000008814) was found as having high-risk mutation at position 27:33105944C>T (p.Gln1341Thr). The protein consists of 1343 aa, and in our case, the aa at position 1341 was affected by the gene mutation. We found no data in the literature regarding its implication in CLJ in cattle and only little information in other species [91]. Regarding the *F13A1* (coagulation factor XIII A chain) gene, the data from the literature does not evidence a linkage with CL/P [92], even if it is mentioned in Cleft GeneDB [60]. The *GSTP1* (glutathione S-transferase P1) was involved in the detoxification of electrophilic compounds by glutathione conjugation, I105V polymorphism in mothers and/or children with/without maternal smoking may increase the risk of CL/P [93].

Two heterozygous insertions were identified for the *CCDC8* gene (ENSBTAG00000018181), the first being 18:g.53693827_53693828insCAGACAA, an insertion of seven nucleotides classified as a frameshift variant, identified at protein level p.Leu374ProfsTer5 (the leucine is the first amino acid changed in position 374 with proline, the length of the shift frame is 5) and the second 18:g53693828-53693829 ins CAGAGGGCAGAGGCCCC, also classified as frameshift variant. In general, frameshift variants have a high impact on the protein, causing in most cases the production of an altered protein 3D structure. The biochemical and cellular functions of this gene are not clearly known, but the protein encoded by the gene acts as a cofactor required for p-53-mediated apoptosis after DNA damage and may also play a role in growth through interactions with the cytoskeletal adaptor protein obscurin-like 1. Deletion of *CCDC8* caused perinatal lethality, intrauterine growth restriction, and placental defects [94]. In humans, the mutation in the 19q13.32 region of *CCDC8* is associated with 3M syndrome dwarfism, a rare inherited disorder characterized by low body weight and short length at birth, short stature as adults, widespread skeletal abnormalities, and unusual facial features [94,95]. The N-terminal and C-terminal of the *CCDC8* proteins are rather conservative across species, suggesting their important functions.

In the *CDH1* gene (ENSBTAG00000015991), the mutation located in position 18:36101086-36101087insT had as a consequence a frameshift, splice region, and intron variant. The *CDH1* gene, which encodes epithelial cadherin, is also very important for the cell–cell adhesions, mobility, and proliferation of epithelial cells, being highly expressed in the frontonasal prominence and in the lateral and medial nasal prominences of human embryos, during critical stages of lip and palate development. Pathogenic variants of CDH1 gene have been implicated in syndromic and non-syndromic cleft lip-palate, as well as in hereditary diffuse gastric cancer [96,97].

Analyzing cleft lip and jaw independently by comparing the association of 13 major risk genes in this case with cleft lip and jaw reported in the literature and in the CleftGeneDB database, we can say that *ACVR1*, *ADGRA2*, *BHMT2*, *BMPR1B*, *CDH1*, *EGF*, *F13A1*, *GSTP1*, *IRF6*, *MMP14*, *MYBPHL*, *PHC2*, except *CCDC8* can be involved in CLJ. As for the hypospadias from the 13 genes, only *CCDC8*, *EGF*, *IRF6*, *CDH1*, and *GSTP1* were reported in literature and in the Gene-Disease Associations dataset [78] as being associated with this anomaly. The protein function of *EGF*, *IRF6*, or *CDH1* in various tissues is to stimulate the growth of epidermal and epithelial tissues or to sustain cell–cell adhesions, so mutation in them can induce congenital abnormalities in the skin in different anatomical regions, as in this case.

Genomic data suggest the involvement of several mutated genes in the critical period of embryonic development of the affected regions. While these non-synonymous homozygous *ADGRA2*, *EGF*, *F13A1*, *IRF6*, and *GSTP1* genes mutation might encode mutant proteins, they do not provide a prediction of the phenotype, but may contribute to more accurate assessments of the orofacial clefts or hypospadias. It is difficult to predict the impact on the protein, because there are many characteristics of proteins that can be modified, such as folding, protein interactions, dynamics, post-translational modifications, or solubility [98]. Knowing the origin of the parents, having no similar cases of products obtained with the same bull, we consider that most likely these mutations are not hereditary, but occurred spontaneously in the embryonic cells of the affected calf. Moreover, other genes, such as *SRD5A2*, *AR*, *DGKK*, *NR5A1*, and *WNT5A*, involved in the steroidogenesis of androgens and in the functionality of hormones, were not identified as having a major risk in this case, but we do not exclude the involvement of endocrine disruptors in the hormone-dependent period of the development of the genital segments external as a possible cause in hypospadias. New research data indicate that DNA methylation sites in the penile foreskin are involved in hypospadias etiology and a potential role of environmental factors that can act through epigenetic alterations and even epigenetic inheritance is suggested [99,100].

Our study expands the gene spectrum toward new genes involved in cleft lip and jaw or in hypospadias, but it is possible that there are other molecular processes coordinated by other genes involved in the presented case that have not been identified. Despite these limitations, the present results provide a basis for further investigation of key genes in cleft lip and jaw or in hypospadias in animals.

## 5. Conclusions

In summary, in our study, we analyzed a unique case of congenital defects in a *Bos taurus* crossbred Piedmontese × Wagyu individual using different paraclinical examinations. The genomic analyses based on whole genome sequencing identified 13 mutations with a predicted high impact on the following overlapped genes: *ACVR1*, *ADGRA2*, *BHMT2*, *BMPR1B*, *CCDC8*, *CDH1*, *EGF*, *F13A1*, *GSTP1*, *IRF6*, *MMP14*, *MYBPHL*, and *PHC2*, of which *ADGRA2*, *EGF*, *F13A1*, *GSTP1*, and *IRF6* showed homozygous mutations, and *BMPR1B* and *CCDC8* had a low or no report in other clinical cases in bovine species regarding CLJ and hypospadias. In veterinary medicine, the genomic data for the two malformations are very rare, although there are numerous clinical reports in the literature about the existence of these congenital pathologies. Therefore, although we cannot specify exactly which of the high-risk genes identified in the study are responsible for the changes identified or if there were environmental factors involved in the appearance of these malformations, we believe that the present study expands the gene spectrum towards new genes involved in cheilognathoschisis or in hypospadias and that the obtained results increase the genetic contribution towards the knowledge of congenital defects in cattle. However, more studies are needed to better understand the specific effects of these genes variants on cheilognathoschisis or on hypospadias in bovine.

## Figures and Tables

**Table 1:** The list of genes from this study found to have high risk consequence.

Gene	Type of Variant	Variant ^a^	Existing Variant ID	Consequence of the Variant	Zygosity ^b^
ACVR1	deletion	2:g38466740delC	NA	frameshift	HET
ADGRA2	substitution	27:g.33105944C>T	rs109168787	stop gained	HOM
BHMT2	substitution	10:g.10153010G>A	rs384114512	splice acceptor	HET
BMPR1B	substitution	6:g.29483476G>A	rs133060728	frameshift	HET
CCDC8	insertion	18:g.53693827_53693828 ins CAGACAA	rs1118258295	frameshift	HET
CCDC8	insertion	18:g53693828-53693829 ins CAGAGGGCAGAGGCCCC	NA	frameshift	HET
CDH1	insertion	18:36101086-36101087insT	NA	frameshift, splice region, intron variant	HET
EGF	substitution	6:g.15450370G>A	rs136048352	Stop gained	HOM
F13A1	deletion	23:48853544delA	NA	frameshift, splice donor region variant	HOM
GSTP1	deletion	29:45444664delG	NA	frameshift	HOM
IRF6	insertion	16:g73512380-73512381insT	NA	frameshift	HOM
MMP14	insertion	10:g21978036-21978037insA	NA	frameshift	HET
MYBPHL	deletion	3:g.34117074delC	rs480413110	frameshift	HET
PHC2	substitution	2:120387448C>T	NA	Stop gained	HET

^a^ Given positions correspond to chromosomes of the ARS-UCD1.2 assembly reference bovine genome. ^b^ HOM—homozygous, HET—heterozygous. NA—not applicable.

## Data Availability

Not applicable.

## Supplementary Materials

The following supporting information can be downloaded at: https://www.mdpi.com/article/10.3390/ani13101709/s1, Figure S1: Average genome coverage: (**a**) Coverage histogram, (**b**) Genome fraction, S1 file: The list of 559 unique bovine genes that were found associated with hypospadias and CLJ in cattle and other mammals, according to literature and the CleftGeneDB database (XLSX); S2 file: The list of SNPs reported by Ensembl VEP as having a high impact on any of the 559 target genes (XLSX); S3 file: The list of SNPs reported by Ensembl VEP as having a moderate impact on any of the 559 target genes (XLSX); S4 file: Gene annotations based on GeneOntology (GO), KEGG, Medical Subject Headings (MeSH), and Reactome, which were found significant after over-representation analyses based on genes with moderate and high impact SNPs (XLSX).

## References

[1] McGeady T.A., Quinn P.J., FitzPatrick E.S., Ryan M.T., Kilroy D., Lonergan P. (2017). Veterinary Embryology.

[2] Moura E., Pimpão C.T. (2017). Cleft Lip and Palate in the Dog: Medical and Genetic Aspects.

[3] Usui K., Tokita M. (2018). Creating diversity in mammalian facial morphology: A review of potential developmental mechanisms. EvoDevo.

[4] Łobodzinska A., Gruszczynska J., Max A., Jan Bartyzel B., Mikula M., Mikula J.I., Grzegrzółka B. (2014). Cleft palate in the domestic dog Canis Lupus Familiaris- etiology, pathophtsiology, diagnosis, prevention and treatment. Acta. Sci. Polonorum. Zootech..

[5] Leslie E.J., Liu H., Carlson J., Shaffer J.R., Feingold E., Wehby G., Laurie C.A., Jain D., Laurie C.C., Doheny K.F. (2016). A Genome-wide Association Study ofS Nonsyndromic Cleft Palate Identifies an Etiologic Missense Variant in GRHL3. Am. J. Hum. Genet..

[6] Jiang R., Bush J.O., Lidral A.C. (2006). Development of the upper lip: Morphogenetic and molecular mechanisms. Dev. Dyn..

[7] Juriloff D.M., Harris M.J. (2008). Mouse genetic models of cleft lip with or without cleft palate. Birth Defects Res. Part A Clin. Mol. Teratol..

[8] Gebuijs I.G.E., Raterman S.T., Metz J.R., Swanenberg L., Zethof J., Van den Bos R., Carels C.E.L., Wagener F.A.D.T.G., Von den Hoff J.W. (2019). Fgf8a mutation affects craniofacial development and skeletal gene expression in zebrafish larvae. Biology Open.

[9] Kazempoor R., Akbarinejad V., Mardjanmehr S.H., Shojaei M., Soroori S., Amini M. (2012). Bilateral cleft lip, jaw, and palate in a female Holstein calf. Comp. Clin. Pathol..

[10] Reinartz S., Hellige M., Feige K., Wenning P., Distl O. (2015). Phenotypic classification of variability of non-syndromic congenital cleft lip and jaw in Vorderwald × Montbéliarde cattle. Acta Vet. Scand..

[11] Koul R. (2007). Describing Cleft Lip and Palate Using a New Expression System. Cleft Palate-Craniofacial J..

[12] Liu Q., Yang M.-L., Li Z.-J., Bai X.-F., Wang X.-K., Lu L., Wang Y.-X. (2007). A Simple and Precise Classification for Cleft Lip and Palate: A Five-Digit Numerical Recording System. Cleft Palate-Craniofacial J..

[13] Guo H.L., Zhao L., Liu Q., Li Z.J., Zhang B., Lu L. (2013). Clinical evaluation of the five-digit numerical recording system for classification of cleft lip and palate deformities. Chin. J. Stomatol..

[14] Saleem K., Zaib T., Sun W., Fu S. (2019). Assessment of candidate genes and genetic heterogeneity in human non syndromic orofacial clefts specifically non syndromic cleft lip with or without palate. Heliyon.

[15] Lupp B., Reinhardt M., Maus F., Hellige M., Feige K., Distl O. (2012). Right-sided cleft lip and jaw in a family of Vorderwald×Montbéliarde cattle. Vet. J..

[16] Shaw S.D., Norman T.E., Arnold C.E., Coleman M.C. (2015). Clinical characteristics of horses and foals diagnosed with cleft palate in a referral population: 28 cases (1988–2011). Can Vet. J..

[17] Oryan A., Shirian S., Samadian M.R. (2011). Congenital craniofacial and skeletal defects with arthrogryposis in two newborn male Holstein Friesian calves. Comp. Clin. Pathol..

[18] Moritomo Y., Tsuda T., Miyamoto H. (1999). Craniofacial Skeletal Abnormalities in Anomalous Calves with Clefts of the Face. J. Vet. Med. Sci..

[19] Stuppia L., Capogreco M., Marzo G., La Rovere D., Antonucci I., Gatta V., Palka G., Mortellaro C., Tetè S. (2011). Genetics of Syndromic and Nonsyndromic Cleft Lip and Palate. J. Craniofacial Surg..

[20] Zucchero T.M., Cooper M.E., Maher B.S., Daack-Hirsch S., Nepomuceno B., Ribeiro L., Caprau D., Christensen K., Suzuki Y., Machida J. (2004). Interferon Regulatory Factor 6 (IRF6) Gene Variants and the Risk of Isolated Cleft Lip or Palate. N. Engl. J. Med..

[21] Dixon M.J., Marazita M.L., Beaty T.H., Murray J.C. (2011). Cleft lip and palate: Understanding genetic and environmental influences. Nat. Rev. Genet..

[22] Sylvester B., Brindopke F., Suzuki A., Giron M., Auslander A., Maas R.L., Tsai B., Gao H., Magee W., Cox T.C. (2020). A Synonymous Exonic Splice Silencer Variant in IRF6 as a Novel and Cryptic Cause of Non-Syndromic Cleft Lip and Palate. Genes.

[23] Robledo R.F., Rajan L., Li X., Lufkin T. (2002). The Dlx5 and Dlx6 homeobox genes are essential for craniofacial, axial, and appendicular skeletal development. Genes Dev..

[24] Hristov K., Stoimenov A. (2020). Hypospadias in small ruminants: A case report. Tradit. Mod. Vet. Med..

[25] Usta Z., Distl O. (2017). Atresia Ani, Hypospadia and Rudimentary External Genitalia in Two German Holstein Calves. Atatürk Üniversitesi Vet. Bil. Derg..

[26] Radostits O., Gay C., Hinchcliff K., Constable P. (2007). Veterinary Medicine, a Textbook of the Diseases of Cattle, Horses, Sheep, Pig and Goats.

[27] Piñeyro-Ruiz C., Serrano H., Pérez-Brayfield M.R., Jorge J.C. (2020). New frontiers on the molecular underpinnings of hypospadias according to severity. Arab. J. Urol..

[28] Chang J., Wang S., Zheng Z. (2020). Etiology of Hypospadias: A Comparative Review of Genetic Factors and Developmental Processes Between Human and Animal Models. Res. Rep. Urol..

[29] Almubarak A.M., Abdelghafar R.M., Gameel A.A., Osman N.M. (2016). Penile Urethral Hypospadias with Two Fistulae and Diverticulum in a Saanen Kid. Case Rep. Vet. Med..

[30] Iannuzzi A., Braun M., Genualdo V., Perucatti A., Reinartz S., Proios I., Heppelmann M., Rehage J., Hülskötter K., Beineke A. (2020). Clinical, cytogenetic and molecular genetic characterization of a tandem fusion translocation in a male Holstein cattle with congenital hypospadias and a ventricular septal defect. PLoS ONE.

[31] Harrison J., Corley K., Kearney C., Mushtaq I. (2018). Single stage urethroplasty for perineal hypospadias in a foal. Equine Vet. Educ..

[32] Abd-El-Hady A.A.A., Mohi Eldin M.M. (2014). Hypospadia and urethral diverticulum in a female pseudohermaphrodite calf. Sch. J. Agric. Vet. Sci..

[33] Hoque M. (2021). A Case Report on Surgical Management of Hypospadias in a Day-Old Goat Kid. Int. J. Livest. Res..

[34] Switonski M., Dzimira S., Aleksiewicz R., Szczerbal I., Nowacka-Woszuk J., Krzeminska P., Deska T., Nizanski W. (2018). Hypospadias Is Not Rare in Dogs: Five New Cases, a Retrospective Study, and a Review of the Literature. Sex. Dev..

[35] Meyers-Wallen V.N. (2012). Gonadal and sex differentiation abnormalities of dogs and cats. Sex. Dev..

[36] Murakami T. (2008). Anatomical Examination of Hypospadias in Cattle. J. Jpn. Vet. Med. Assoc..

[37] Kumi-Diaka J., Osori D.I.K. (1979). Perineal hypospadias in two related bull calves, a case report. Theriogenology.

[38] van der Zanden L.F.M., van Rooij I.A.L.M., Feitz W., Franke B., Knoers N.V.A.M., Roeleveld N. (2012). Aetiology of hypospadias: A systematic review of genes and environment. Hum. Reprod. Update.

[39] Raghavan R., Romano M.E., Karagas M.R., Penna F.J. (2018). Pharmacologic and Environmental Endocrine Disruptors in the Pathogenesis of Hypospadias: A Review. Curr. Environ. Health Rep..

[40] Bouty A., Ayers K.L., Pask A., Heloury Y., Sinclair A.H. (2015). The Genetic and Environmental Factors Underlying Hypospadias. Sex. Dev..

[41] Chen Z.-Z., Gao Y.-Q., Xie H., Huang Y.-C., Chen F., Lei Y.-P., Zhu Y.-Q. (2022). Transcription factors dysregulated in three complex birth defects datasets. Reprod. Dev. Med..

[42] Murashima A., Kishigami S., Thomson A., Yamada G. (2015). Androgens and mammalian male reproductive tract development. Biochim. Biophys. Acta (BBA) Gene Regul. Mech..

[43] Chen Y., Yu H., Pask A.J., Fujiyama A., Suzuki Y., Sugano S., Shaw G., Renfree M.B. (2018). Hormone-responsive genes in the SHH and WNT/β-catenin signaling pathways influence urethral closure and phallus growth. Biol. Reprod..

[44] Govers L.C., Phillips T.R., Mattiske D.M., Rashoo N., Black J.R., Sinclair A., Baskin L.S., Risbridger G.P., Pask A.J. (2019). A critical role for estrogen signaling in penis development. FASEB J..

[45] Baskin L., Cao M., Sinclair A., Li Y., Overland M., Isaacson D., Cunha G.R. (2020). Androgen and estrogen receptor expression in the developing human penis and clitoris. Differentiation.

[46] Chen Z., Lin X., Wang Y., Xie H., Chen F. (2020). Dysregulated expression of androgen metabolism genes and genetic analysis in hypospadias. Mol. Genet. Genom. Med..

[47] Yucel S., Cavalcanti A.G., Desouza A., Wang Z., Baskin L.S. (2003). The effect of oestrogen and testosterone on the urethral seam of the developing male mouse genital tubercle. BJU Int..

[48] Vottero A., Minari R., Viani I., Tassi F., Bonatti F., Neri T.M., Bertolini L., Bernasconi S., Ghizzoni L. (2011). Evidence for Epigenetic Abnormalities of the Androgen Receptor Gene in Foreskin from Children with Hypospadias. J. Clin. Endocrinol. Metab..

[49] Ohsako S., Aiba T., Miyado M., Fukami M., Ogata T., Hayashi Y., Mizuno K., Kojima Y. (2018). Expression of Xenobiotic Biomarkers CYP1 Family in Preputial Tissue of Patients with Hypospadias and Phimosis and Its Association with DNA Methylation Level of SRD5A2 Minimal Promoter. Arch. Environ. Contam. Toxicol..

[50] Andrews S. Babraham Bioinformatics. https://www.bioinformatics.babraham.ac.uk/projects/fastqc/.

[51] Wood D.E., Lu J., Langmead B. (2019). Improved metagenomic analysis with Kraken 2. Genome Biol..

[52] O’Leary N.A., Wright M.W., Brister J.R., Ciufo S., Haddad D., McVeigh R., Rajput B., Robbertse B., Smith-White B., Ako-Adjei D. (2016). Reference sequence (RefSeq) database at NCBI: Current status, taxonomic expansion, and functional annotation. Nucleic Acids Res..

[53] Null D.J., Vanraden P.M., Rosen B.D., O’Connell J.R., Bickhart D.M. (2019). Using the ARS-UCD1.2 reference genome in U.S. evaluations. Interbull Bull..

[54] Li H. (2013). Aligning sequence reads, clone sequences and assembly contigs with BWA-MEM. arXiv.

[55] Howe K.L., Achuthan P., Allen J., Allen J., Alvarez-Jarreta J., Amode M.R., Armean I.M., Azov A.G., Bennett R., Bhai J. (2021). Ensembl 2021. Nucleic Acids Res..

[56] van der Auwera G.A., O’Connor B.D. (2020). Genomics in the Cloud.

[57] McLaren W., Gil L., Hunt S.E., Riat H.S., Ritchie G.R.S., Thormann A., Flicek P., Cunningham F. (2016). The Ensembl Variant Effect Predictor. Genome Biol..

[58] R Core Team A Language and Environment for Statistical Computing. R Foundation for Statistical Computing, Vienna, Austria. https://www.R-project.org/.

[59] Fernández N., Pabon J., Ayala P., Perez J., Ortiz A.M., Zarante I. (2018). Description of a novel variant in the MAMLD1 gene in isolated hypospadias. Urol. J..

[60] Xu H., Yan F., Hu R., Suzuki A., Iwaya C., Jia P., Iwata J., Zhao Z. (2021). CleftGeneDB: A resource for annotating genes associated with cleft lip and cleft palate. Sci. Bull..

[61] Morgan M., Shepherd L. AnnotationHub: Client to Access AnnotationHub Resources. R Package Version 3.4.0. https://bioconductor.org/packages/release/bioc/html/AnnotationHub.html.

[62] The Gene Ontology Consortium (2019). The Gene Ontology Resource: 20 years and still GOing strong. Nucleic Acids Res..

[63] Kanehisa M., Araki M., Goto S., Hattori M., Hirakawa M., Itoh M., Katayama T., Kawashima S., Okuda S., Tokimatsu T. (2008). KEGG for linking genomes to life and the environment. Nucleic Acids Res..

[64] Lipscomb C.E. (2000). Medical Subject Headings (MeSH). Bull Med. Libr. Assoc..

[65] Jassal B., Matthews L., Viteri G., Gong C., Lorente P., Fabregat A., Sidiropoulos K., Cook J., Gillespie M., Haw R. (2020). The reactome pathway knowledgebase. Nucleic Acids Res..

[66] Yu G., Wang L.-G., Han Y., He Q.-Y. (2012). clusterProfiler: An R package for comparing biological themes among gene clusters. OMICS.

[67] Yu G. (2018). Using meshes for MeSH term enrichment and semantic analyses. Bioinformatics.

[68] Yu G., He Q.-Y. (2016). ReactomePA: An R/Bioconductor package for reactome pathway analysis and visualization. Mol. BioSystems.

[69] Pagès H., Carlson M., Falcon S., Li N. Bioconductor. https://bioconductor.org/packages/release/bioc/html/AnnotationDbi.html.

[70] Yan F., Dai Y., Iwata J., Zhao Z., Jia P. (2020). An integrative, genomic, transcriptomic and network-assisted study to identify genes associated with human cleft lip with or without cleft palate. BMC Med. Genom..

[71] Krzemińska P., D’Anza E., Ciotola F., Paciello O., Restucci B., Peretti V., Albarella S., Switonski M. (2019). Polymorphisms of MAMLD1, SRD5A2 and AR Candidate Genes in Seven Dogs (78,XY.; SRY-Positive) Affected by Hypospadias or Cryptorchidism. Sex. Dev..

[72] Switonski M., Payan-Carreira R., Bartz M., Nowacka-Woszuk J., Szczerbal I., Colaço B., Pires M.A., Ochota M., Nizanski W. (2012). Hypospadias in a Male (78,XY.; SRY-Positive) Dog and Sex Reversal Female (78,XX.; SRY-Negative) Dogs: Clinical, Histological and Genetic Studies. Sex. Dev..

[73] Miyado M., Nakamura M., Miyado K., Morohashi K.-I., Sano S., Nagata E., Fukami M., Ogata T. (2012). Mamld1 deficiency significantly reduces mRNA expression levels of multiple genes expressed in mouse fetal Leydig cells but permits normal genital and reproductive development. Endocrinology.

[74] Peycelon M., Mansour-Hendili L., Hyon C., Collot N., Houang M., Legendre M., Chabaud M., Bouvier M.D., Audry G., Amselem S. (2017). Recurrent Intragenic Duplication within the NR5A1 Gene and Severe Proximal Hypospadias. Sex. Dev..

[75] Song Y., Chen W., Zhu B., Ge W. (2022). Disruption of Epidermal Growth Factor Receptor but Not EGF Blocks Follicle Activation in Zebrafish Ovary. Front. Cell Dev. Biol..

[76] Miettinen P.J., Chin J.R., Shum L., Slavkin H.C., Shuler C.F., Derynck R., Werb Z. (1999). Epidermal growth factor receptor function is necessary for normal craniofacial development and palate closure. Nat. Genet..

[77] Paiva K.B.S., Maas C.S., Santos P.M.d., Granjeiro J.M., Letra A. (2019). Extracellular Matrix Composition and Remodeling: Current Perspectives on Secondary Palate Formation, Cleft Lip/Palate, and Palatal Reconstruction. Front. Cell Dev. Biol..

[78] Rouillard A.D., Gundersen G.W., Fernandez N.F., Wang Z., Monteiro C.D., McDermott M.G., Ma’ayan A. (2016). The harmonizome: A collection of processed datasets gathered to serve and mine knowledge about genes and proteins. Database.

[79] Noda K., Mishina Y., Komatsu Y. (2016). Constitutively active mutation of ACVR1 in oral epithelium causes submucous cleft palate in mice. Dev. Biol..

[80] Sanchez-Duffhues G., Williams E., Goumans M.-J., Heldin C.-H., ten Dijke P. (2020). Bone morphogenetic protein receptors: Structure, function and targeting by selective small molecule kinase inhibitors. Bone.

[81] Li L., Wang Y., Lin M., Yuan G., Yang G., Zheng Y., Chen Y. (2013). Augmented BMPRIA-Mediated BMP Signaling in Cranial Neural Crest Lineage Leads to Cleft Palate Formation and Delayed Tooth Differentiation. PLoS ONE.

[82] Gupta C., Siegel S., Ellis D. (1991). The role of EGF in testosterone-induced reproductive tract differentiation. Dev. Biol..

[83] Chen T., Li Q., Xu J., Ding K., Wang Y., Wang W., Li S., Shen Y. (2007). Mutation screening of BMP4, BMP7, HOXA4 and HOXB6 genes in Chinese patients with hypospadias. Eur. J. Hum. Genet..

[84] de la Garza G., Schleiffarth J.R., Dunnwald M., Mankad A., Weirather J.L., Bonde G., Butcher S., Mansour T.A., Kousa Y.A., Fukazawa C.F. (2013). Interferon Regulatory Factor 6 Promotes Differentiation of the Periderm by Activating Expression of Grainyhead-Like 3. J. Investig. Dermatol..

[85] Girousi E., Muerner L., Parisi L., Rihs S., von Gunten S., Katsaros C., Degen M. (2021). Lack of IRF6 Disrupts Human Epithelial Homeostasis by Altering Colony Morphology, Migration Pattern, and Differentiation Potential of Keratinocytes. Front. Cell Dev. Biol..

[86] Knight A.S., Schutte B.C., Jiang R., Dixon M.J. (2006). Developmental expression analysis of the mouse and chick orthologues of IRF6: The gene mutated in Van der Woude syndrome. Dev. Dyn..

[87] Jugessur A., Rahimov F., Lie R.T., Wilcox A.J., Gjessing H.K., Nilsen R.M., Nguyen T.T., Murray J.C. (2008). Genetic variants in IRF6 and the risk of facial clefts: Single-marker and haplotype-based analyses in a population-based case-control study of facial clefts in Norway. Genet. Epidemiol..

[88] Zhang M., Zhang J., Zhao H., Ievlev V., Zhong W., Huang W., Cornell R.A., Lin J., Chen F. (2020). Functional Characterization of a Novel IRF6 Frameshift Mutation From a Van Der Woude Syndrome Family. Front. Genet..

[89] Gurramkonda V.B., Syed A.H., Murthy J., Lakkakula B.V.K.S. (2018). IRF6 rs2235375 single nucleotide polymorphism is associated with isolated non-syndromic cleft palate but not with cleft lip with or without palate in South Indian population. Braz. J. Otorhinolaryngol..

[90] Kousa Y.A., Moussa D., Schutte B.C. (2017). IRF6 expression in basal epithelium partially rescues Irf6 knockout mice. Dev. Dyn..

[91] Isono K.-I., Fujimura Y.-I., Shinga J., Yamaki M., O-Wang J., Takihara Y., Murahashi Y., Takada Y., Mizutani-Koseki Y., Koseki H. (2005). Mammalian polyhomeotic homologues Phc2 and Phc1 act in synergy to mediate polycomb repression of Hox genes. Mol. Cell Biol..

[92] Hecht J.T., Wang Y., Connor B., Daiger S.P., Blanton S.H. (1993). Nonsyndromic cleft lip and palate: No evidence of linkage to HLA or factor 13A. Am. J. Hum. Genet..

[93] Krapels I.P.C., Raijmakers-Eichhorn J., Peters W.H.M., Roelofs H.M.J., Ras F., Steegers-Theunissen R.P.M., the Eurocran Gene–Environment Interaction G. (2008). The I105V polymorphism in glutathione S-transferase P1, parental smoking and the risk for nonsyndromic cleft lip with or without cleft palate. Eur. J. Hum. Genet..

[94] Wang P., Yan F., Li Z., Yu Y., Parnell S.E., Xiong Y. (2019). Impaired plasma membrane localization of ubiquitin ligase complex underlies 3-M syndrome development. J. Clin. Investig..

[95] Hanson D., Murray P.G., O’Sullivan J., Urquhart J., Daly S., Bhaskar S.S., Biesecker L.G., Skae M., Smith C., Cole T. (2011). Exome sequencing identifies CCDC8 mutations in 3-M syndrome, suggesting that CCDC8 contributes in a pathway with CUL7 and OBSL1 to control human growth. Am. J. Hum Genet.

[96] Selvanathan A., Nixon C.Y., Zhu Y., Scietti L., Forneris F., Moreno Uribe L.M., Lidral A.C., Jezewski P.A., Mulliken J.B., Murray J.C. (2020). CDH1 Mutation Distribution and Type Suggests Genetic Differences between the Etiology of Orofacial Clefting and Gastric Cancer. Genes.

[97] Frebourg T., Oliveira C., Hochain P., Karam R., Manouvrier S., Graziadio C., Vekemans M., Hartmann A., Baert-Desurmont S., Alexandre C. (2006). Cleft lip/palate and CDH1/E-cadherin mutations in families with hereditary diffuse gastric cancer. J. Med. Genet.

[98] Katsonis P., Wilhelm K., Williams A., Lichtarge O. (2022). Genome interpretation using in silico predictors of variant impact. Hum. Genet..

[99] Richard M.A., Sok P., Canon S., Nembhard W.N., Brown A.L., Peckham-Gregory E.C., Ton M., Ehli E.A., Kallsen N.A., Peyton S.A. (2020). Altered mechanisms of genital development identified through integration of DNA methylation and genomic measures in hypospadias. Sci. Rep..

[100] Kaefer M., Rink R., Misseri R., Winchester P., Proctor C., Ben Maamar M., Beck D., Nilsson E., Skinner M.K. (2023). Role of epigenetics in the etiology of hypospadias through penile foreskin DNA methylation alterations. Sci. Rep..

