# Supplementary material for: Simultaneous Occurrence of Hypospadias and Bilateral Cleft Lip and Jaw in a Crossbred Calf: Clinical, Computer Tomographic, and Genomic Characterization
Source: Animals (Basel). 2023 May 22;13(10):1709. doi: 10.3390/ani13101709 (PMC10215116; doi:10.3390/ani13101709)
Supplement: Supplementary file 1 [file animals-13-01709-s001.zip › Supplementary Materials_Animals_2023_Cristina Paul.pdf]

Simultaneous occurrence of hypospadias and bilateral cleft lip and jaw in a crossbred calf: clinical, computer tomographic and genomic characterization

Simona Marc[†1,2], Alexandru Eugeniu Mizeranschi[†3], Cristina Paul[4*], Gabriel Otavă[1,2], Jelena Savici[1], Bogdan Sicoe[1], Iuliu Torda[1,2,] Ioan Huțu[1,2], Călin Mircu[1,2,], Daniela Elena Ilie[3], Mihai Carabaş[5], and Oana Maria Boldura[†1,2]

[1] University of Life Sciences ''King Mihai I'' from Timisoara, Faculty of Veterinary Medicine, Calea Aradului 119, 300645 Timisoara, Romania; simona.marc@usab-tm.ro (S.M.); gabrielotava@usab-tm.ro (G.O); jelenasavici@usab-tm.ro (J.S.); bogdan.sicoe@usab-tm.ro (B.S.); iuliu.torda@usab-tm.ro (I.T.); ioanhutu@usab-tm.ro (I.H.); calinmircu@usab-tm.ro (C.M.); oanaboldura@usab-tm.ro (O.B.);

[2] University of Life Sciences ''King Mihai I'' from Timisoara, Research Institute for Biosecurity and Bioengineering"; simona.marc@usab-tm.ro (S.M.); gabrielotava@usab-tm.ro (G.O); iuliu.torda@usab-tm.ro (I.T.); ioanhutu@usab-tm.ro (I.H.); calinmircu@usab-tm.ro (C.M.); oanaboldura@usab-tm.ro (O.B.)

[3] The Molecular Research Department, Research and Development Station for Bovine Arad, Bodrogului Street, 32, 310059 Arad, Romania, alex.mizeranschi@gmail.com (A.E.M.), danailie@animalsci-tm.ro (D.E.I.);

[4] Politehnica University Timisoara, Faculty of Industrial Chemistry and Environmental Engineering, Department of Applied Chemistry and Engineering of Organic and Natural Compounds, Carol Telbisz 6, 300001 Timisoara, Romania; cristina.paul@upt.ro (C.P.);

[5] Politehnica University of Bucharest, Faculty of Automatic Control and Computer Science, Splaiul Independenţei 313, 060042 Bucharest, Romania, mihai.carabas@cs.pub.ro (M.C.);

* Correspondence: cristina.paul@upt.ro;

† These authors contributed equally to the work

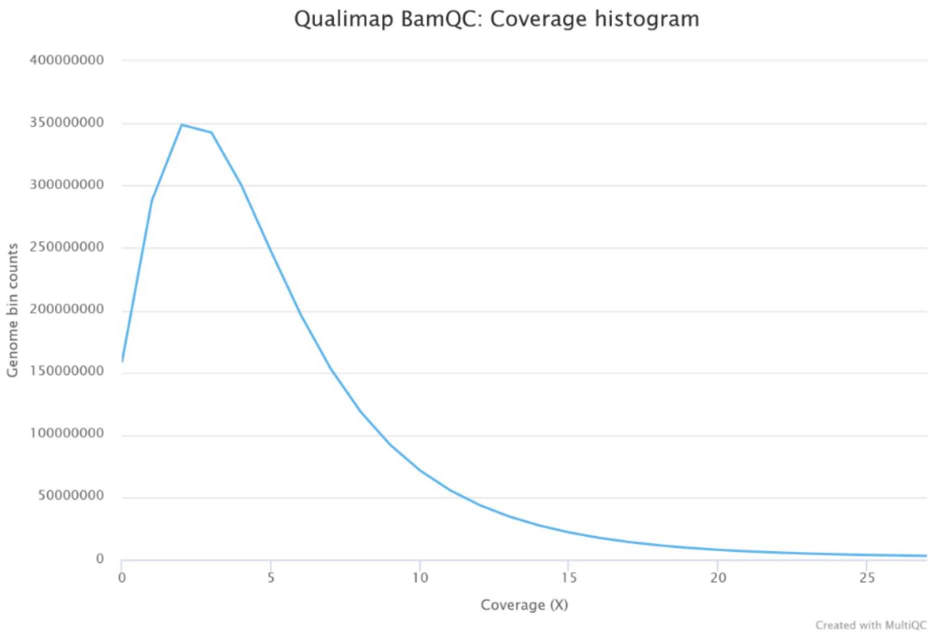

(a)

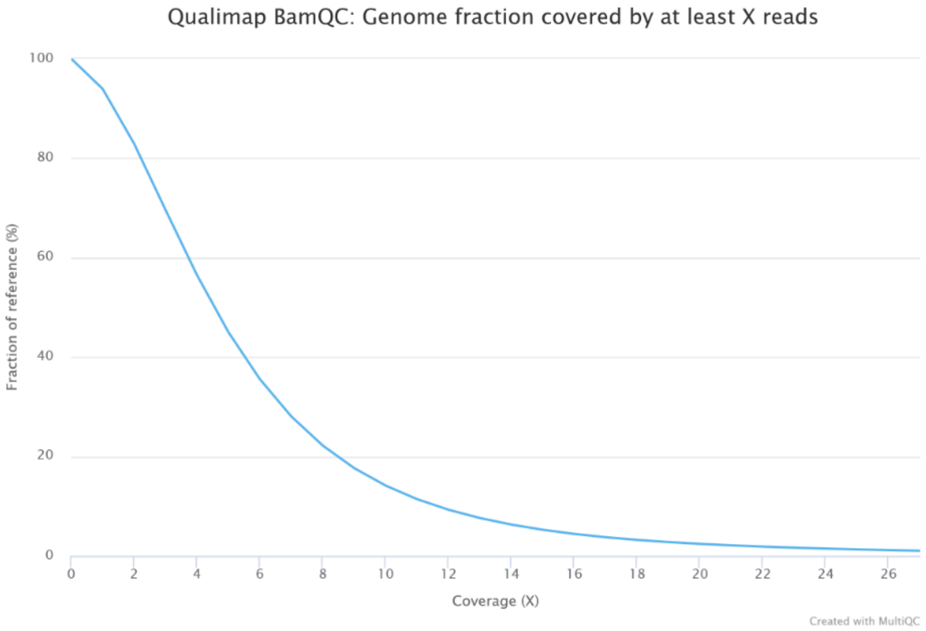

(b)

Figure S1. Average genome coverage: (**a**) Coverage histogram, (**b**) Genome fraction.
